# Evaluating the Adiabatic Invariants in Magnetized Plasmas Using a Classical Ehrenfest Theorem

**DOI:** 10.3390/e25111559

**Published:** 2023-11-18

**Authors:** Abiam Tamburrini, Sergio Davis, Pablo S. Moya

**Affiliations:** 1 Departamento de Física, Facultad de Ciencias, Universidad de Chile, Santiago 8370459, Chile; 2 Research Center in the Intersection of Plasma Physics, Matter and Complexity (P2mc), Comisión Chilena de Energía Nuclear, Casilla 188-D, Santiago 8320000, Chile; sergdavis@gmail.com; 3 Departamento de Física, Facultad de Ciencias Exactas, Universidad Andres Bello, Santiago 8370136, Chile

**Keywords:** Ehrenfest theorem, magnetized plasmas, non-equilibrium statistical mechanics

## Abstract

In this article, we address the reliance on probability density functions to obtain macroscopic properties in systems with multiple degrees of freedom as plasmas, and the limitations of expensive techniques for solving Equations such as Vlasov’s. We introduce the Ehrenfest procedure as an alternative tool that promises to address these challenges more efficiently. Based on the conjugate variable theorem and the well-known fluctuation-dissipation theorem, this procedure offers a less expensive way of deriving time evolution Equations for macroscopic properties in systems far from equilibrium. We investigate the application of the Ehrenfest procedure for the study of adiabatic invariants in magnetized plasmas. We consider charged particles trapped in a dipole magnetic field and apply the procedure to the study of adiabatic invariants in magnetized plasmas and derive Equations for the magnetic moment, longitudinal invariant, and magnetic flux. We validate our theoretical predictions using a test particle simulation, showing good agreement between theory and numerical results for these observables. Although we observed small differences due to time scales and simulation limitations, our research supports the utility of the Ehrenfest procedure for understanding and modeling the behavior of particles in magnetized plasmas. We conclude that this procedure provides a powerful tool for the study of dynamical systems and statistical mechanics out of equilibrium, and opens perspectives for applications in other systems with probabilistic continuity.

## 1. Introduction

Statistical mechanics provides a theoretical framework and a toolbox that gives us a deep understanding and detailed description of the collective behavior of particles in physical systems. One of the main advantages of statistical mechanics lies in its ability to relate observable macroscopic properties to the microscopic properties of individual particles. By using probabilistic and statistical methods, we can greatly simplify the analysis of complex systems, and obtain general rules applicable to a wide range of conditions. Through this connection, we can understand how macroscopic properties emerge from the behavior of particles at the microscopic level [1]. Furthermore, statistical mechanics offers a unified approach to analyze equilibrium and non-equilibrium systems. It allows us to study not only systems at thermodynamic equilibrium but also non-equilibrium systems [2,3,4,5]. This capability is especially relevant for modeling complex phenomena in nature [6,7,8]. Treating a non-equilibrium system is still an open task [9]. However, we have seen approximations based on the fundamental theory of statistical mechanics, such as the principle of maximum entropy [10], that, from first principles and information theory [11], we can infer probability distribution for the state of the system, which is most likely given certain restrictions and the information available. This approach allows us to describe the dynamics of non-equilibrium systems by providing a coherent picture of how they evolve over time, thus providing a clear prescription for constructing models in non-equilibrium statistical mechanics [12,13]. These models have been explored to understand the time evolution of dynamical systems [14,15,16,17], leaving us with general rules applicable to this type of system [18].

Although we can find plasma in an equilibrium state, usually when collisions are scarce, a plasma is typically a system out of equilibrium with a high number of degrees of freedom [19]. In the study of plasmas, there are various approaches from non-equilibrium statistical mechanics that allow an adequate description of these complex systems. Two prominent approaches are non-extensive statistical mechanics [20] and superstatistics [21], both aimed at addressing the features that emerge when plasma particles are far from thermodynamic equilibrium. Non-extensive statistical mechanics represents a theoretical framework that quantifies the distance from equilibrium in these systems through non-extensive entropy. This approximation generalizes the Boltzmann–Gibbs entropy, which has traditionally been used to describe systems in thermodynamic equilibrium. However, plasmas often have dynamic and structural properties that differ significantly from a classical steady state. In this sense, non-extensive statistical mechanics offers a more suitable way to characterize systems with non-trivial fluctuations and correlations, which is common in plasmas due to their turbulent behavior and non-linear transport properties [22,23,24,25]. On the other hand, superstatistics is another approach that is not based on generalizing the Boltzmann–Gibbs entropy, but instead proposes the existence of other parameters that follow a distribution different than Boltzmann [21,26,27]. These parameters can be considered effective temperatures in different cells of the plasma, where each cell has a temperature distribution that deviates from a global temperature in a possible thermodynamic equilibrium. In this view, the plasma particles do not experience a single, global temperature, but rather ‘feel’ different temperatures depending on the cell they belong to. This non-trivial characteristic of temperature in the plasma has important implications for the dynamics and macroscopic properties of the system. These advanced theoretical tools contribute to a better understanding of the physical processes in plasmas [28,29,30,31].

In general, from the point of view of statistical mechanics, these systems are described by partial differential equations (PDEs) for the time-dependent probability distribution function of the system. With this, it is possible to calculate the macroscopic properties of the system using the expectation value of its microscopic properties. The plasmas are described by Vlasov’s PDE [32]. This equation comes from Liouville’s theorem, where the Hamiltonian involves kinetic terms and the interaction of charged particles with electromagnetic fields [33]. There is no general analytical solution to this problem for arbitrary electromagnetic fields for the probability density function, and the numerical methods are extremely expensive in terms of computational cost, as they require the discretization of space and time with a grid size as small as the desired resolution, which depends of the highest frequency of modes of oscillation in the plasma, typically the frequency associated with the electrons [34].

To study the plasma macroscopic properties, we have developed new non-equilibrium statistical mechanics techniques, in particular, what has been called the ‘Ehrenfest procedure’ due to its resemblance to Ehrenfest’s theorem in quantum mechanics [35]. The procedure allows the construction of particular differential equations associated with an arbitrary and time-dependent macroscopic property *w*. Using two fundamental theorems of statistical mechanics, the conjugate variable theorem (CVT) and the fluctuation–dissipation theorem (FDT) [18,36], it is possible to relate the derivatives in time and space of the probability density function with the derivatives in time and space of an arbitrary time-dependent macroscopic property. Therefore, employing this feature, we can eliminate the explicit dependence of the probability density function, ρ, on the original PDE, obtaining a particular PDE associated with an arbitrary macroscopic property *w*. Moreover, in Ref. [35], the procedure was applied to the Vlasov equation to formulate new equations for the general evolution of arbitrary fluctuations in collisionless plasmas.

On the other hand, it is well known that in classical mechanics, whenever there is a system with periodic movements, the action integral ∮pdq, with *p* and *q* the generalized variables in the phase space, taken over a period, is a constant of motion [37]. If under small perturbations in the system this quantity remains constant, then we call it adiabatic invariant. These quantities are important in plasma physics and help us simplify the answer in cases involving complex motion. The study of adiabatic invariants allows us to identify and characterize properties and relations that remain constant during the temporal evolution of a system. It is possible to predict significant quantities and magnitudes of systems in different stages [38]. It allows studying conditions in which the system is stable or maintains an equilibrium state [39]. At the particle scale, it allows the characterization of their movement and the understanding of their dynamics in environments where they interact with electromagnetic fields, such as particles trapped in the Earth’s magnetic field [40], giving rise to Earth’s radiation belts. In particular, for particles trapped in a dipole magnetic field, there are three invariants associated with three types of movement, namely: gyromotion around magnetic field lines, bouncing between two magnetic mirror points, and orbital motion around the Earth due to the curvature drift [41,42,43]. In this work, we make use of the Ehrenfest procedure applied to the Vlasov equation to study the adiabatic invariants in a magnetized collisionless plasma.

We have organized this document as follows. First, in Section 2, we show the three adiabatic invariants associated with the three motions of the particles under a magnetic field, and we present the expression that the Ehrenfest procedure determines for the time evolution of each of these quantities. Then, in Section 3, we present the numerical result obtained from a test particle simulation and show the correlation between the analytical expression shown in Section 2 and the numerical result. Finally, in Section 4, we summarize our findings and present the main conclusions.

## 2. Ehrenfest Procedure in Adiabatic Invariants

As already mentioned, in a previous study [35], it was shown that by applying the Ehrenfest procedure to the Vlasov equation, it is possible to derive a dynamical PDE for an arbitrary observable, equivalent to the Vlasov equation. Following Ref. [35], we define the Ehrenfest operator given by

(1) $$\mathrm{Eh}\left[•\right]=\langle \frac{\partial •}{\partial t}+v\cdot \partial_{r}•+\frac{F}{m}\cdot \partial_{v}•\rangle ,$$

where the brackets correspond to the expectation value, defined as follows:

(2) $$W\left(t\right)=\langle w(r,v,t)\rangle =\int drdv\;\rho \left(r,v\right)w\left(r,v,t\right)$$

Therefore, the PDE for the arbitrary observable corresponds to the equation that follows the form

(3) $$\frac{\partial }{\partial t}\langle w\rangle =\mathrm{Eh}\left[w\right]\;,$$

namely,

(4) $$\frac{\partial }{\partial t}\langle w\rangle =\langle \frac{\partial w}{\partial t}\rangle +\langle v\cdot \partial_{r}w\rangle +\langle \frac{F}{m}\cdot \partial_{v}w\rangle ,$$

where the notation Eh[w] represents the expression derived from the Ehrenfest procedure on an arbitrary observable, equal to the temporal evolution of this observable *w*.

Here, we are interested in the study of adiabatic invariants of particles trapped in a magnetic field. For example, in the case of the Earth’s radiation belt, there are three main motions of the particles due to their interaction with the approximate dipole magnetic field of the Earth [44]. Given this approximation, it is clear to see that due to the curvature of the field, there will be different drifts that give rise to these movements [37]. The invariants, their associated motion, and the Ehrenfest procedure applied to each of them are described below.

- First Invariant.

The first invariant of magnetic moment is associated with the Larmor gyration, and it is given by the expression,
  (5) $$\mu =\frac{mv_{⊥}^{2}}{2B}.$$
  Considering the magnetic moment as an observable μ=w, we replace the right hand of Equation (5) in Equation (4):
  $$\frac{\partial }{\partial t}\langle \mu \rangle =\mathrm{Eh}\left[\mu \right]\;.$$
  Therefore, we obtain
  (6) $$\begin{array}{c} \frac{\partial }{\partial t}\langle \mu \rangle =-[\langle \frac{mv_{⊥}^{2}}{2}\frac{\partial }{\partial t}\left(\frac{1}{B}\right)\rangle +\langle \frac{mv_{⊥}^{2}}{2}v\cdot \nabla_{r}\left(\frac{1}{B}\right)\rangle ], \end{array}$$
  where Equation (6) corresponds to the Ehrenfest expression for the time variation of the magnetic moment. The first addend of this expression refers to the temporal variation of the magnetic field. If the magnetic field has fast variations, it can cause direct variation in the magnetic moment of a particle. If the change in the magnetic field is fast enough, it could even induce electric currents, which in turn would affect its magnetic moment. The second addend refers to the existence of drifts. Given a curvature in the magnetic field, the Larmor orbits can expand or contract accordingly, and the particles may lose or gain energy in the transverse direction, affecting the average magnetic moment.

- Second Invariant.

The second adiabatic invariant, known as the longitudinal invariant, is associated with the periodic motion along the magnetic field line for particles trapped between two magnetic mirror points. It is possible to study this motion indirectly through the pitch angle between the particle velocity and the magnetic field direction, given by the expression
  (7) $$\cos\left(\theta \right)=\frac{B\cdot v}{\left|B||v\right|}.$$
  Considering the pitch angle as an observable cos(θ)=w, we replace the right hand of Equation (7) in Equation (4):
  (8) $$\frac{\partial }{\partial t}\langle \cos(\theta )\rangle =\mathrm{Eh}\left[\cos\left(\theta \right)\right]=\langle v\cdot \left(\hat{v}\cdot \nabla_{r}\right)\hat{B}\rangle .$$
  Thus, Equation (8) shows that when a charged particle moves in a nonuniform magnetic field, it experiences a magnetic force that acts perpendicular to its velocity and the magnetic field. We can demonstrate this non-uniformity in Equation (8), given by the gradient of the projected field on the direction of movement of the particle. This force can change the direction of motion of the particle, which in turn affects the angle between the velocity and the magnetic field lines, that is, the pitch angle.

- Third Invariant.

Finally, the third adiabatic invariant, called magnetic flux invariant, is associated with the nonuniform B field drift, which if it is not time-dependent, should expect close trajectories; therefore, we can study it indirectly through the average (squared) radial distance of a particle radio particles. Then, making the observable $w=r^{2}$, we replace in Equation (4), obtaining
  (9) $$\frac{\partial }{\partial t}\langle r^{2}\rangle =\mathrm{Eh}\left[r^{2}\right]=\langle v\cdot r\rangle .$$
  In this case, it is more direct to see that the variation of the average radial distance of each particle will depend on whether it moves in the radial direction. For this reason, the variation of the third invariant depends directly on the projection of the velocity of the particles in the radial direction.
  The set of equations that form Equations (6), (8), and (9) corresponds to the analytical results for the temporal evolution of the quantities that allow us to study the movements to which the three adiabatic invariants are associated in this system. To verify these three expressions obtained using the Ehrenfest procedure, both sides of Equations (6), (8), and (9) will be compared using the data extracted from test particle simulations.

## 3. Numerical Results for Testing the Ehrenfest Procedure in Adiabatic Invariants

**Figure 1:**
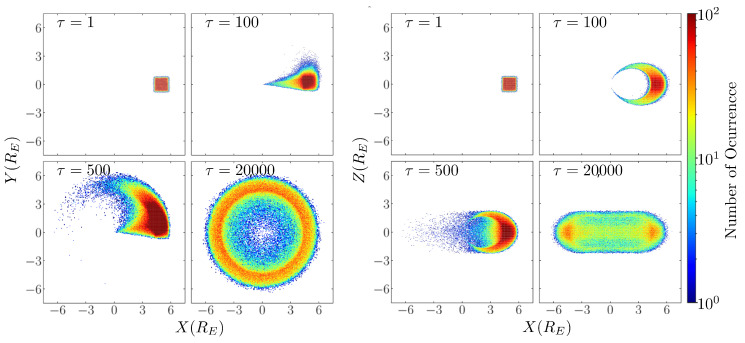
Particle trajectories in the $\hat{x}$-$\hat{y}$ plane in the left panel and $\hat{x}$-$\hat{z}$ plane in the right panel, in four different time steps: τ=1, τ=100, τ=500, and τ= 20,000.

In order to verify the theoretical expressions derived in Section 2, we developed a test particle simulation [45] code written in the C++ programming language. In this kind of simulation, the particles follow the trajectories prescribed by the electromagnetic field, do not interact with each other, as in particle-in-cell simulations [34,46,47], and do not influence the dynamics of electromagnetic fields. Our objective was to simulate the outer Earth’s radiation belt conditions. The particles were trapped by the Earth’s magnetic field, which was considered a dipole magnetic field of the form

(10) $$B\left(r\right)=\frac{3\left(m\cdot r\right)-r^{2}m}{r^{5}},$$

where r is particle position, and m is the magnetic moment that, in our case, was considered in the $\hat{z}$ direction. In setting the initial conditions, we adopted a Maxwellian distribution comprising $10^{5}$ particles. The velocities were stochastically assigned according to this distribution, employing the Box–Muller method and employing a thermal velocity that aligns with energy levels on the order of keV, normalized with respect to the rest energy of electrons. The particles were injected in a small cube near the magnetic equator at a radial distance of $4.5\pm 0.5\;R_{E}$, with $R_{E}$ being the Earth’s radius. The simulation was run for $1.4\times 10^{6}$ time steps Δτ=0.1, where $\tau =c\;t/R_{E}$ represents the normalized time, and *c* is the speed of light. To obtain the velocity and position of the particle, the Boris algorithm was used, which is based on Liouville’s theorem, and preserves energy by construction without being symplectic [48]. The first result corresponds to the particle trajectories, where we notice that the particles follow the expected behavior given the conditions. In Figure 1, we can see the evolution of the particle distribution in four different moments during the simulation. Three of them (τ= 1, 100, and 500) correspond to the first moments of the simulation in which the radiation belt is still forming, and the last one (τ= 20,000) corresponds to a later stage in which the belt has been completed.

**Figure 2:**
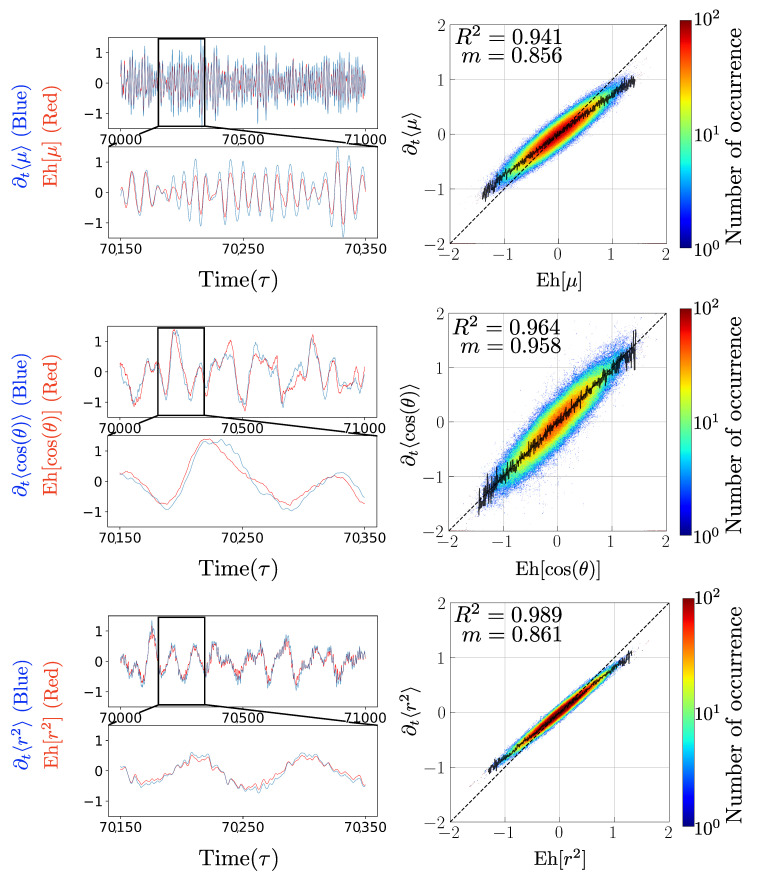
Application of the Ehrenfest procedure to particles trapped in a dipole magnetic field. Left: time series of the left-hand (blue) and right-hand (red) sides of Equation (4) using data extracted from the simulation. From top to bottom, each panel corresponds to the Ehrenfest procedure applied to the magnetic moment, pitch angle, and radial distance, respectively. Right: correlations between left- and right-hand sides of each relation. In each scatter plot, the solid black line represents the maximum in each column, and black dashed lines represent the tendency and identity, respectively.

Since the spatial particle distribution reaches a stationary state, that is, once the radiation belt has been formed after approximately 10,000 time steps, and the particles follow their periodic motion, we build the time series for each quantity. On the one hand, we build the time derivative of the observable, and on the other, we build the time series for the quantities that the Ehrenfest procedure indicates to determine the time evolution of the observable; that is, using the simulation we construct and compare the left and right sides of the Equations (6), (8), and (9), and to construct the expectation values of the different quantities, we calculate the arithmetic average over all the particles for a fixed time, given by the discrete data that we can extract from the simulation. In the left column of Figure 2, from top to bottom, time series corresponding to both sides of Equations (6), (8), and (9) are plotted in red and blue. In addition, the right column of the same figure shows scatter plots of the same quantities (magnetic moment, pitch angle, and average radial distance), so we compare them with each other with respect to the trend line that represents Slope 1 (black dashed line). We calculate the Pearson correlation coefficient between quantities and plot both sides of the Equations (6), (8), and (9) to be able to see the level of correlation between both sides of the Ehrenfest equation for each adiabatic invariant. Finally, we made a linear fit to each data set and calculated the slope of the fit in each case, which is shown in Figure 2 along with the Pearson correlation coefficient.

In the complete dynamics of the system, we have that the three adiabatic invariants are on different time scales. For example, the first invariant is on the order of the motion of the cyclotron around the magnetic field line, given by the Larmor frequency, and the flux invariant is given by the precession of particles around the Earth. This means that the equations of motion must be solved over a wide range of time scales.

Since the time series are constructed based on different parameters and fluctuate on different time scales, the data of each series were normalized before being treated for construction, This allows the order of fluctuation of each time series to be the same, eliminating the error caused by the centered derivative by having to make a finite difference between quantities that are in the order of $10^{-25}$, such as the magnetic moment. The time series show small differences that are best evident in the lower window of each time series, where we approach a specific time interval.

We can see that the three invariants fluctuate on different time scales, and each quantity presents a different noise in its curve. For example, for the magnetic moment, it is possible to distinguish its distinctive oscillation period with respect to the equilibrium value being at least 10 times smaller than the other two quantities. On the other hand, for the average radius of the orbit, it is possible to distinguish a period of oscillation around the equilibrium value, but at the same time, it contains noise in the curve, which occurs to a lesser extent for the pitch angle. From this second invariant, we can see that the fluctuation follows the same behavior and, as expected, presents a better Pearson correlation coefficient of 0.964 compared to the correlation coefficient of 0.941 obtained for the magnetic moment.

We also note that by the construction of the second invariant, this is already normalized and is where we find the best slope m=0.958, and as expected, it is better than the slope of the magnetic moment m=0.856, which, in principle, fluctuates in the order previously mentioned. Finally, for the third adiabatic invariant, we note that although the curve contains more noise, this expression presents the best Pearson correlation coefficient of 0.980, and in the zoom window, we distinguish that the time series also follows the same behavior.

Although the analytical expression suggests that the relationship between the temporal evolution of the adiabatic invariant should be 1 to 1 with the expression determined by the Ehrenfest procedure, it is expected to obtain small differences between the time series that represent both sides of the Equations obtained using the Ehrenfest procedure. This is because it only approximates the behavior of a Vlasov system since, by using the numerical simulation, we discretized the space. The numerical methods are in fact finite differences; that is, we are working only with a section of the phase space and not with the entire phase space as a Vlasov system would follow. In Figure 2, these differences and errors are manifested by less than one correlation coefficient and also in that the slopes of the fits for each quantity are below the 1-to-1 relationship suggested by the analytical expression. Despite this, we notice in the time series windows that each quantity follows the same behavior and presents a very good correlation and a slope very close to Slope 1.

## 4. Conclusions

In this article, we have explored the applicability of the Ehrenfest procedure for the study of adiabatic invariants in magnetized plasmas. The reliance on probability density functions to obtain macroscopic properties in systems with a high number of degrees of freedom and the expensive techniques, both theoretical (such as canonical transformations in the Vlasov equation) and computational to solve the problem, motivated us to propose a procedure that would allow obtaining information about the system in a less expensive way than conventional methods, that is, the Ehrenfest procedure. This procedure is based on general rules such as the conjugate variable theorem [18], valid for states arbitrarily far from equilibrium, covering specific cases such as hypervirial [49] relations in statistical mechanics and the expression of temperature in the microcanonical ensemble, as derived by Rugh and generalized by Rickayzen [50,51]. Its validity remains independent of considerations about ergodicity or a priori equality of probabilities for microstates in a thermodynamic system, even when the existence of such a system is uncertain. Instead, it holds as long as we base our reasoning on incomplete information, given in the form of expected values. This theorem can serve as an alternative tool for the estimation of parameters in probability distributions that present sufficient statistics, seen from the perspective of frequentist statistics.

We have demonstrated the application of our ‘Ehrenfest procedure’ to the Vlasov equation for collisionless plasmas. We use the general relation in equation (4), which gives the time evolution of any macroscopic property in a Vlasov system. From Ehrenfest’s general relation, we have derived the partial differential equation for the study, both direct and indirect, of the three adiabatic invariants: magnetic moment, longitudinal invariant, and magnetic flux, which we show in Equations (5), (7), and (9). We compare the theory with the numerical simulations, and the results suggest a good agreement between the theory and the numerical simulations for the magnetic moment, the average radius of the orbit, and the pitch angle, which suggests that the procedure is suitable for these observables. In addition, the increase in the statistics of the problem since the simulation approaches a Vlasov system, and we only have a part of the phase space, together with the fact that the numerical methods used are of finite difference, can give rise to differences and errors in the observed correlations, which explains the fluctuations and noises present in the curves of the different quantities analyzed. Due to the aforementioned, with this procedure, we were able to demonstrate the equality given by the analytical expression in the numerical simulation data up to the simulation precision. It is also worth mentioning that although Boris’s algorithm is based on construction on Liouville’s theorem, it is not symplectic; therefore, it does not preserve the volume in the phase space. Thus, although it is expected that there is some numerical error, we observe that the analytical expressions are mostly confirmed by the simulations up to numerical precision.

**Figure 3:**
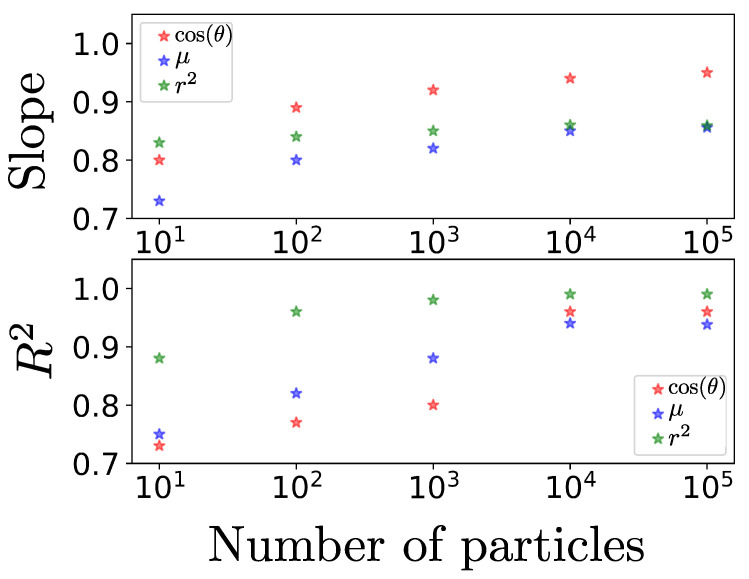
The upper panel shows the change in the slope of the linear fit for each quantity as we increase the particle statistics of the problem in steps of an order of magnitude. The lower panel shows the variation of the Pearson correlation coefficient by increasing the number of particles. The error associated with each data is on the order of 0.02% on average; therefore, the error bars are not distinguishable on the scale of the figure.

We have observed an increase in the Pearson correlation coefficient and the slope of the linear fit as we increase the statistics collected from the problem. Specifically, by increasing the number of particles in the simulation by an order of magnitude, the correlation coefficient increases significantly, as shown in Figure 3, where we also see that it seems to stabilize between $10^{4}$ and $10^{5}$. Here, we present the case of 10${}^{5}$ particles, as it is the smallest number that leads to meaningful results without the use of unnecessary numerically expensive simulations. In summary, we have shown that the Ehrenfest procedure is a general useful tool, applicable to any system following a continuity equation for probability, such as the Vlasov equation in collisionless plasmas. Given these advantages, applications seem to come naturally. For example, in a plasma modeled by the Fokker–Planck equation, it would be possible to have expressions for observables with dependencies on the drift and diffusion coefficient. It remains for future work to show that this procedure is useful to analyze the case in which the magnetic field intensity is suddenly altered in this system of trapped particles, simulating a geomagnetic storm. In such a case, it should be possible to violate the third adiabatic invariant and expect the particles to undergo radial diffusion [44] that, in principle, may be estimated using this procedure. In general, this procedure allows one to quickly obtain equations of dynamic evolution for particular properties and, therefore, seems to be a powerful addition to the study of out-of-equilibrium plasmas or dynamical systems in general.

## Data Availability

The data presented in this study are openly available in Zenodo at https://doi.org/10.5281/zenodo.8411207.

## Abbreviations

The following abbreviations are used in this manuscript:

PDE	partial differential equations
CVT	conjugate variable theorem
FDT	fluctuation–dissipation theorem
